# The Role of Medical Imaging in the Recharacterization of Mild Traumatic Brain Injury Using Youth Sports as a Laboratory

**DOI:** 10.3389/fneur.2015.00273

**Published:** 2016-01-19

**Authors:** Thomas M. Talavage, Eric A. Nauman, Larry J. Leverenz

**Affiliations:** ^1^School of Electrical and Computer Engineering, Weldon School of Biomedical Engineering, Purdue University, West Lafayette, IN, USA; ^2^Department of Basic Medical Sciences, School of Mechanical Engineering, Weldon School of Biomedical Engineering, Purdue University, West Lafayette, IN, USA; ^3^Department of Health and Kinesiology, Purdue University, West Lafayette, IN, USA

**Keywords:** concussion, traumatic brain injury, functional MRI, magnetic resonance imaging, magnetic resonance spectroscopy, subconcussive

## Abstract

The short- and long-term impact of mild traumatic brain injury (TBI) is an increasingly vital concern for both military and civilian personnel. Such injuries produce significant social and financial burdens and necessitate improved diagnostic and treatment methods. Recent integration of neuroimaging and biomechanical studies in youth collision-sport athletes has revealed that significant alterations in brain structure and function occur even in the absence of traditional clinical markers of “concussion.” While task performance is maintained, athletes exposed to repetitive head accelerations exhibit structural changes to the underlying white matter, altered glial cell metabolism, aberrant vascular response, and marked changes in functional network behavior. Moreover, these changes accumulate with accrued years of exposure, suggesting a cumulative trauma mechanism that may culminate in categorization as “concussion” and long-term neurological deficits. The goal of this review is to elucidate the role of medical imaging in recharacterizing TBI, as a whole, to better identify at-risk individuals and improve the development of preventative and interventional approaches.

## Introduction

On Friday, October 20, 2006, Cody Lehe, a senior at Frontier High School in Indiana, participated in a high school football game, much as he had each autumn since he was a Freshman. On this particular day, Cody experienced dizziness and blurred vision following a severe head-to-head blow during the game, but insisted he was fine and continued playing. Concerned about the blow, Cody’s coaches encouraged his parents to monitor him over the weekend. After experiencing some sensitivity to light Friday evening, Cody reported no symptoms the remainder of the weekend, keeping his persistent headache to himself. Finally, on Tuesday morning, he admitted to his parents that he was still experiencing a headache from the previous week’s game. Cody was promptly taken to the local hospital and treated according to the standard-of-care for possible concussion symptoms – he underwent a computed tomography (CT) exam to determine if he had experienced a fractured skull or had a subdural hematoma. For both potentially serious outcomes, the CT exam was negative. As a result, Cody was sent home and informed that he could return to football practice that day provided his headache had subsided. Later that afternoon, following a typical scrimmage play early in the practice, Cody collapsed and became unresponsive. As detailed in *The Impact of Cody Lehe* (1), Cody struggles to this day with short-term memory loss and the inability to walk or live independently.

**Figure 1:**
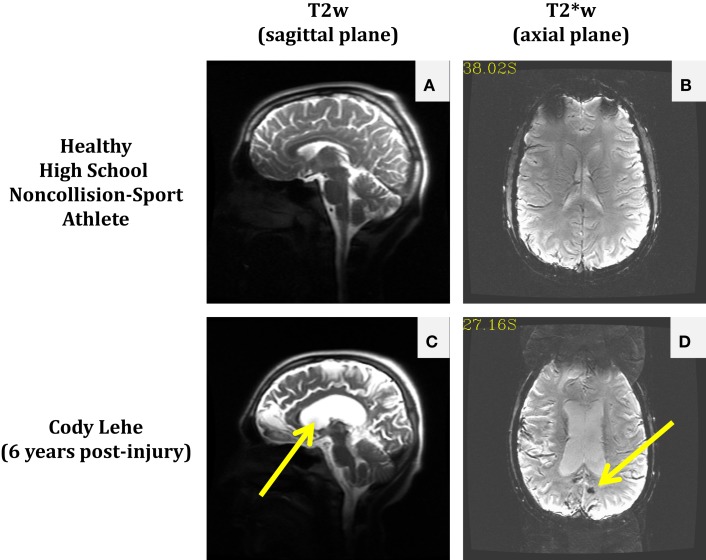
**Comparison of anatomical and susceptibility-weighted images in a healthy non-collision-sport high school athlete (*top*) and a former athlete who experienced significant neurological injury while in high school, Cody Lehe (*bottom*)**. T2-weighted images (sagittal plane) are presented for the control athlete **(A)** and Cody **(C)** to illustrate abnormal post-injury anatomy (e.g., enlarged ventricles indicated by the arrow). T2*-weighted images (axial plane) are presented for the control athlete **(B)** and Cody **(D)** to illustrate the presence of an abnormal lesion in the post-injury case (arrow).

The permanent alteration to Cody’s life was not inherently due to receiving a particularly damaging second blow (e.g., so-called “Second Impact Syndrome”), but rather the consequence of structural damage that was not readily visible in the imaging modality associated with the standard-of-care. Magnetic resonance imaging (MRI) scans of Cody’s brain taken in 2012 (see Figure 1) suggest that either an aneurysm was present or he experienced a slow leak in his cerebral arteries at the time of his initial injury. In either case, this weak point became a significant bleed under the physical exertion associated with his return to full contact participation on the following Tuesday, with the final insult possibly provided by the ordinary tackle made on his final play.

The inability to identify some forms of damage with the standard-of-care diagnostic procedures (as in Cody’s case) serves as a beacon, signaling the need for development of novel detection and diagnostic protocols for brain injury. This point should further be recognized as imperative, given the well-documented underreporting of concussions and symptoms by athletes (2–7). Further, it is already recognized that athletes who experience a second concussion in close temporal proximity to a first injury are likely to experience more severe symptoms, emphasizing the importance of effective early injury detection.

It is critical to recognize that increasing the number of health-care professionals at the field (and thereby appreciably raising the cost of youth athletics) is unlikely to be a successful solution to the problem of detection. In spite of regular observation by certified athletic trainers and team physicians, college football athletes participating in a survey subsequent to the 2012 season reported being diagnosed with a concussion only one out of every seven times (14%) that they suspected they were so injured (8). In the case of high school athletes and younger – where the presence of qualified health-care professionals on the sideline is at best sporadic – such numbers strongly indicate that symptom-based identification of brain injuries is not an effective means of protecting the neurological health of athletes.

The idea that symptoms are insufficient as a means of identifying brain injury has previously been well-documented by work from our research group and that of several others. These works document that neurophysiologic alterations can affect brain behavior – without producing readily diagnosable symptoms – during task performance (9, 10), resting-state analysis (11–13), and breath-hold challenge (14). Structural changes have also been observed in asymptomatic athletes exposed to repetitive subconcussive blows, in both white (15, 16) and gray matter (17, 18). Further, the biochemistry of these athletes has been found to vary over the course of exposure to many acceleration events to the head (19, 20). Given the numbers reported by Baugh et al. (8), and that all of these changes have been observed in what are otherwise asymptomatic subjects, it is clear that the definition of “concussion” is not sufficiently broad. Rather it appears that this definition merely covers one extreme of a broad range of injuries.

Clearly, traumatic brain injury (TBI) is more than a problem with football. The short- and long-term consequences of TBI are increasingly vital concerns for both military and civilian personnel. Approximately 5.3 million Americans currently live with long-term disabilities as a result of some form of TBI [see Ref. (21)]. It has been estimated that 22% of military casualties (22) suffer from TBI and many more experience post-traumatic stress disorder (PTSD). Among civilians, conservative estimates indicate that, in 2003, there were 1.5 million cases of mTBI in the United States, resulting in 51,000 deaths, 290,000 hospitalizations, and over 1 million emergency room visits. The incidence of TBI is especially high in children under the age of 14, with nearly half a million cases per year (21).

From the above discussion, it is apparent that advanced medical imaging has a significant role to play in shaping how brain injury is diagnosed and subsequently treated. The present lack of ready, convenient access to such technology may limit its long-term potential as a broad-based diagnostic tool (i.e., it is not financially practical to place diagnostic imaging equipment proximal to every athletic field). However, imaging must continue to serve as a “gold standard” for the assessment and validation of more portable diagnostic alternatives, which may ultimately allow a larger portion of the population to engage in activities such as collision-based sports while also facilitating better brain health. This study has been carried out in accordance with the Declaration of Helsinki and was approved by the Human Research Protection Program, Biomedical IRB.

## Brain Injury – An Engineering View

Whether it be concussion or any other form of TBI, direct physical disruption of the cellular membrane is the commonly conceived form of brain damage. However, alterations in metabolic homeostasis may occur even when the membrane is not disrupted (23, 24). Such disruptions may occur directly or indirectly (e.g., due to changes in the local ionic environment) as a result of the application of external mechanical forces. Changes in metabolic homeostasis will necessarily induce responses from the cells that seek to restore the ionic balances associated with “healthy” function. If we recognize these responses as efforts at “repair,” we must also recognize that the alterations to metabolism represent “injury” even if they do not represent *permanent* structural or biologic alterations.

Coupling the recognition that injury is more appropriately defined as any incident that has altered the metabolism of the cells with the massively complex nature of the brain, it is readily apparent that the brain will not respond to injury in a monolithic manner. While we commonly attribute observable symptoms (e.g., pain, inability to function) to injury, it is not unprecedented in the medical domain to recognize that the onset of injury is not inherently accompanied by symptoms (cf., cumulative strain injuries, such as stress fractures or Carpal tunnel syndrome, in which the symptoms are predictive of only a portion of the physical damage). In other words, injury to one particular locale in the brain – be it a neuron, an axonal tract, or even the vasculature – need not necessarily produce failure (i.e., “symptoms”) in most, or even many, attempts to produce an output (i.e., a behavior) that would typically involve that locale.

**Figure 2:**
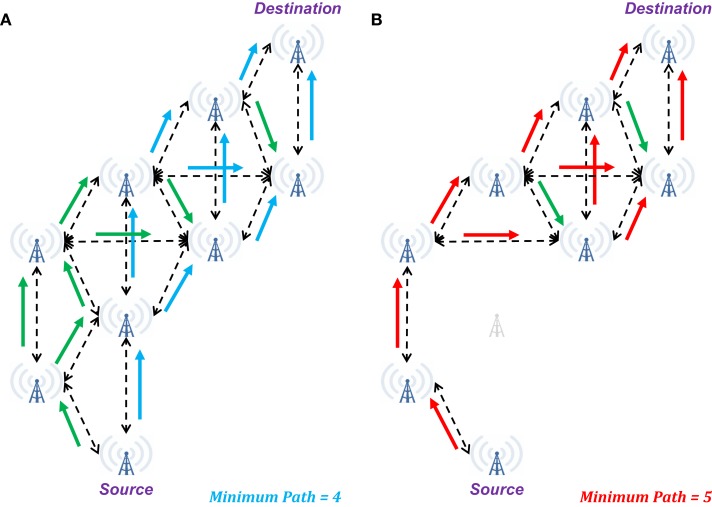
**Illustration of changes in network connectivity using an *ad hoc* communications network as a model of brain connectivity**. **(A)** When fully connected, a wide range of possible pathways can result in successful transmission of a signal from the Source to the Destination in four steps. Additionally, multiple intermediate towers may receive simultaneous inputs that may be necessary to induce subsequent retransmission of the signal. **(B)** When a single node in the network is eliminated, the minimum pathway extends to five steps from Source to Destination, with a risk of several intermediate nodes only receiving a single input. In the context of “summation in place,” these single input cases may be insufficient for the message to reach the Destination. In the context of “summation in time,” the longer duration to reach the Destination may preclude expected behavior at this final node. Green arrows indicate additional valid transmission pathways that are not a part of the minimum length path.

Ignoring the function of the glia and the mechanisms of neurovascular coupling, the billions of neurons in the brain may be modeled as a distributed system, with perhaps the most accessible current model being that of an *ad hoc* communications network (e.g., Figure 2). In such a network, successful transmission of information from Source to Destination involves reception of sufficient information routed through multiple parallel pathways (e.g., the unidirectional arrows in Figure 2A). Note that some packets transmitted from the Source may fail to reach the Destination (e.g., they may be dropped due to network traffic or poor reception), but in most cases, no individual packet is necessary for successful transmission (i.e., each generally contains too little information to affect overall transmission if lost). Additionally, some sequentially related packets may arrive out of order (due to pathways of different lengths and delays), but provided, they arrive within a given window of time, they will be appropriate reordered to successfully reconstruct the information.

From a neuronal perspective, the two *ad hoc* communication network cases described above are comparable to the concepts of summation in place and summation in time. In the former, post-synaptic potentials (excitatory or inhibitory) produce the desired hyper- or hypo-polarization only if they arrive *en masse* to a small region of the receiving neuron, so a single “dropped packet” is unlikely to affect the outcome. For the latter, the desired polarization is achieved only if the inputs arrive with sufficient synchrony to accumulate a change in polarization before homeostatic mechanisms have restored the resting potential.

Therefore, in the brain, the pathway from Source to Destination is sufficiently intact for “healthy” behavior so long as the necessary signal may reach the Destination with sufficient strength (e.g., summation in place) and within a requisite window of time (e.g., summation in time). Should enough of these pathways become weakened (e.g., reduced membrane integrity, dissociation of the myelin sheath) or intervening synapses become dysfunctional (i.e., stochastic firing becomes less likely), the situation becomes like that of an *ad hoc* communications network with a dead node – packets may either cease to reach their destination or be delayed (as illustrated in Figure 2B). In either case, the expected signal may not be received at the Destination with sufficient fidelity for successful transmission of information. In the context of the brain, this means that the Destination now does not receive the expected inputs, and its behavior will become erratic – specifically it may now fail to produce or inhibit an action potential.

However, it should be noted that many cases exist in both *ad hoc* communications networks and the brain where specific towers or nodes represent a “final common pathway” for information in a given transmission pathway, and the loss of these key elements will be sufficient to preclude an expected output for a given input. Therefore, recognizing the varied levels of importance for nodes within the brain makes it clear that “symptoms” may be generated from accumulations of multiple injuries or even from a single blow that just happens to damage a critical pathway.

Therefore, most low-level injury (“fatigue”) to the brain should not, in fact, be expected to produce observable symptoms (“failures”). Rather, it would be anticipated that the gradual accrual of brain injury would produce more effortful completion of tasks, or greater metabolic demand. Such is supported by the history of concussion (e.g., symptoms only under physical stress or difficulty with concentration on hard tasks).

## Structural Health Monitoring – An Engineering Solution

For decades, engineers have recognized that dangerous levels of damage can accumulate in a material or structure without being visible to the naked eye. There are numerous examples of sudden, catastrophic failures of engineered systems from damage that occurred inside a material component. For example, experiences associated with the development and growth of microfractures around square windows in the De Havilland Comet in the 1950s prompted the use of flight logs to identify the number of decompression–compression cycles experienced by an airframe, with both maintenance and removal from service dictated by the number of such cycles.

More importantly, failures such as the De Havilland Comet spurred development of methods for non-destructive evaluation including acoustic emissions, eddy current testing of welds, infrared and thermal measurement systems, and vibration analysis. Engineers also employed methods pioneered in medicine such as radiographs and CT, ultrasound, and MRI. These tools ultimately became the foundation for the field of structural health monitoring (SHM), which has dramatically improved safety in the airline and automotive industries, military, and the food industry.

It is reasonable to propose that SHM may be effectively applied to enhance brain health in cases where neurotrauma is a potential outcome. Such an approach necessitates that one interpret TBI as a condition where an individual may accrue symptom-inducing injury in a gradual (“subconcussive”), or singularly traumatic (“concussive”) manner. This general description of injury is familiar to most in the engineering domain, particularly in fields associated with materials that undergo repeated stresses of varying levels that may “fatigue” from use, and which risk catastrophic “failure” should that accumulation of fatigue (here, analogous to “injury”) not be detected.

**Figure 3:**
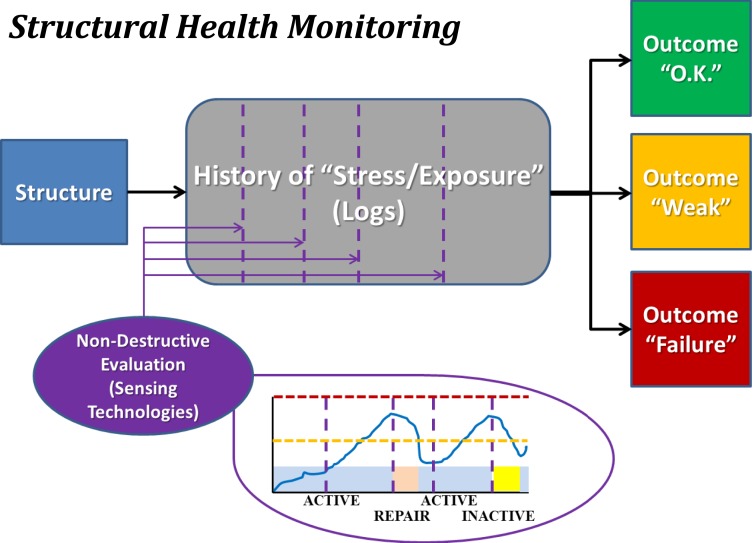
**Block diagram illustrating the concept of structural health monitoring (SHM)**. The structure of interest is monitored for usage over time, and periodically assessed using non-destructive evaluation methodologies. Each such assessment permits categorization of the structure as being healthy (“O.K.”), altered (“Weak”), or severely damaged (“Failure”). Note that depicted images are intended only as schematic representations of data and/or associated analyses.

Structural health monitoring (see Figure 3) incorporates prospective monitoring and predictive modeling of the stress applied to a structure and the internal damage experienced by the materials that comprise the structure. SHM is used to provide guidelines as to when a particular structure should undergo preventative maintenance to mitigate accrued damage or be removed from service to preclude catastrophic failure. Everyday mechanical systems governed by SHM that could otherwise experience notable catastrophic failures include airplanes and bridges. Airplanes undergo frequent non-destructive evaluation, typically using ultrasound, to confirm that microfracture growth has remained within expected and acceptable bounds. Bridges are assessed with tools such as strain gages to confirm that they may continue to be loaded at “normal” levels (i.e., those for which they were designed), or if measures must be taken either to reduce loading or to repair/replace portions of the structure. In both cases, projections may be made as to the success of bearing future normative loads, while also permitting detection – ideally prior to catastrophic failure (e.g., Aloha Airlines Flight 243 and the I-35W bridge in Minneapolis) – of abnormal loading that may accelerate the failure process.

The SHM approach may be adapted from the realm of mechanical systems to the domain of neuroscience, expressly not only to improve characterization of brain injury but also to provide a means for enhancing detection, prevention, and intervention.

## Application of SHM to Brain Injury – The Purdue Neurotrauma Group Study

Beginning in 2009, the Purdue Neurotrauma Group (PNG) initiated a longitudinal, prospective study of high school collision-sport and non-collision-sport athletes, following the general design of SHM, with MRI serving as the primary modality of non-destructive evaluation.

**Figure 4:**
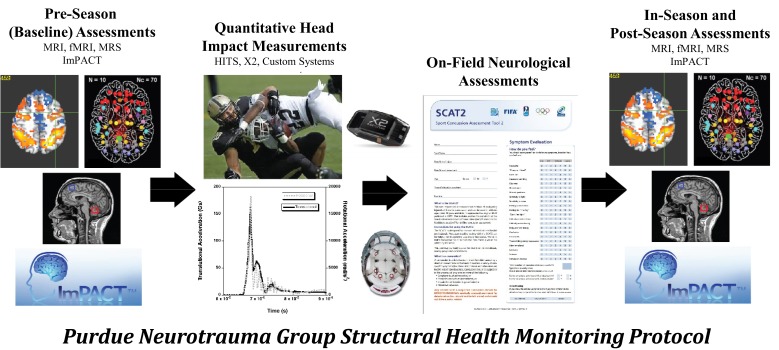
**The Purdue Neurotrauma Group (PNG) study has been conducted on collision- and non-collision-sport athletes at the high school and collegiate levels, using a structural health monitoring framework (see Figure 3)**. Athletes are continually monitored using head- or helmet-based telemetry, allowing tracking of exposure to acceleration events. Athlete neurophysiology is assessed using both neurocognitive and neuroimaging methodologies up to five times per season: once prior to exposure, at least once during exposure, and at least once subsequent to exposure. Outcomes of these assessments may be compared within an athlete or against a reference population. Over the first 6 years of the study, 380 athlete-seasons were evaluated with 1080 neuroimaging assessments.

Under the applied SHM protocol (Figure 4), collision-sport athletes undergo “Baseline” neurologic assessment using neuroimaging and neurocognitive testing prior to exposure to stresses (i.e., before collision-based practices commence) are monitored using the same tools during the period of exposure (i.e., “In-Season”) and are then evaluated again following the period of exposure (i.e., “Post-Season”). In addition to the neuroimaging and neurocognitive assessments, athletes have additionally been evaluated for their exposure to physical stress through head- or helmet-based telemetry systems.

Assessments on non-collision-sport athletes (i.e., high school athletes with no history of participation in interscholastic collision-based sports) provide key information on which to base the evaluation of collision-sport athletes. First, longitudinal assessment of non-collision-sport athletes provide measures of test–retest reliability for the MRI and neurocognitive methodologies used. Second, and perhaps most importantly, these assessments provide a reference for how a “healthy” brain should appear. This reference permits the consequences of recent and prior injuries to be identified in peers by deviations from the healthy population range. Further, this reference population permits identification of abnormal measurements even in the absence of pre-injury (i.e., Baseline or In-Season) assessments, though the attribution of causation cannot be strongly made.

A key element of the SHM approach that has contributed significantly to the findings of the PNG study is the acquisition of pre-injury assessments for each studied individual. These assessments, taken at Baseline and (potentially) In-Season prior to the development of symptoms, enable deviant measurements that existed *prior* to an injury (i.e., diagnosis of a concussion) to be ruled out as sequelae of the identified TBI. As such, these changes may be appropriately attributed either to the “natural” state of the individual in question (when present at Baseline), or as a pre-cursor change (when arising at asymptomatic In-Season assessments) and provide the most clear evidence that subconcussive injury has occurred to which the aberrant observations should be attributed as sequelae. SHM evaluations thus provide a longitudinal history of exposure and neurophysiologic and neurocognitive responses to that exposure, allowing these athletes to be classified at any measurement as “Healthy” (no deviant measurements or symptoms), “Altered” (deviant measurements in the absence of symptoms), or “Injured” (symptoms with or without deviant measurements) in both a relative (when compared to themselves) or absolute (when compared to non-collision-sport peers) sense.

It is in this manner that the PNG study has repeatedly demonstrated that youth athletes participating in collision sports (specifically boys’ football and girls’ soccer) experience low-level brain injury that alters neurophysiologic function at a substantially higher rate than is predicted by the observed frequency of symptomatic injury. Additionally, the PNG study has repeatedly observed that the degree of subconcussive (i.e., asymptomatic) injury increases with sustained exposure to head acceleration events that produce torque and stress on brain tissue. Results from a variety of evaluation measurements are summarized below not only to provide a sense of the scope of evidence for subconcussive injury but also to illustrate how the SHM framework permits tests with potentially low specificity to contribute meaningfully to the detection and characterization of brain injury.

The non-destructive evaluation conducted on the athletes, as noted previously, involves both neurocognitive and neuroimaging assessments that comprise more than 15 individual measurements of brain health and behavior at each time-point. Neuroimaging measurements have included: functional assessments using working memory task-based functional MRI (fMRI), resting-state fMRI (rs-fMRI), and a breath-hold challenge during fMRI to assess cerebrovascular reactivity (CVR); structural assessments using T1-weighted (T1w) imaging for morphometric analysis of gray matter health, diffusion-weighted imaging (DWI) to measure white matter health, and T2*-weighted (T2*w) imaging for assessment of microhemorrhages [e.g., Ref. (25)] – a quick approximation to susceptibility-weighted imaging (SWI); and MR spectroscopy (MRS) of the dorsolateral prefrontal cortex (DLPFC) and the primary motor cortex (M1), examining in each volume of interest the concentrations of total creatine-containing compounds (tCr), total choline-containing compounds (tCho), inositol (Ins), *N*-acetylaspartate (NAA), and glutamate + glutamine (Glx). Neurocognitive testing is accomplished using the Immediate Post-Concussion Assessment and Cognitive Testing battery, ImPACT™. Changes in these measures are typically evaluated in the context of the accumulated exposure to head acceleration events, as reported by the telemetry systems – *helmet-based:* Head Impact Telemetry System (HITS™) (Simbex, LLC); *head-based:* xPatch (X2Biosystems, Inc.).

### Neurocognitive Evaluation

Neurocognitive testing, with ImPACT™ or similar computer-based tools, has become a common component of concussion care for athletes at the collegiate and professional levels and has additionally been greatly encouraged in recent years for youth athletes. While the majority of the relatively short-duration (i.e., 25 min or less) neurocognitive tests are documented to have poor test–retest reliability (26) and relatively high false positive rates (27), the common usage of these tools for confirmation of the diagnosis of a concussion and their common role in return-to-play decision-making motivate inclusion in a SHM approach to brain injury. In the PNG study, neurocognitive testing has revealed so-called false positive rates in athletes undergoing exposure to repeated head collision events (28, 29) that are appreciably higher than observed in non-collision-sport controls (27), and these abnormal observations persist well beyond the end of the period of exposure (29). While such tools may have a relatively low specificity, the results are consistent with the hypothesis that brain function is altered as a result of repeated exposure to head acceleration events, even if this alteration does not lead to readily identifiable symptoms.

### Functional MRI Evaluation

**Figure 5:**
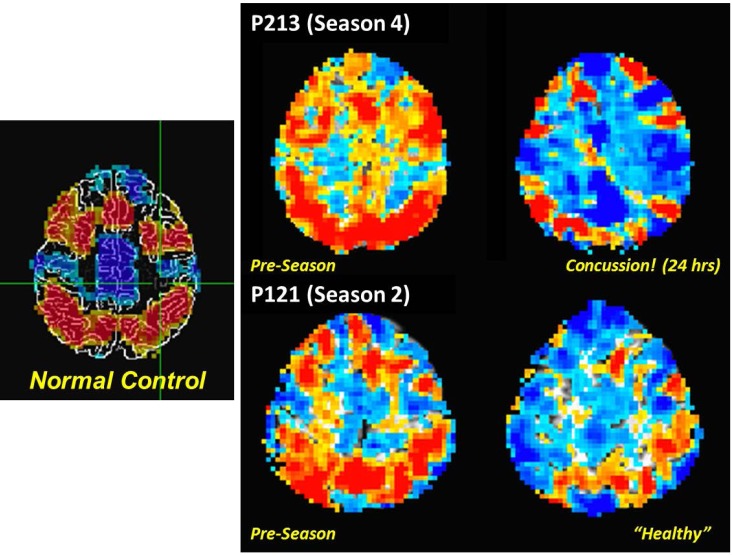
**Comparison of fMRI measurements of “verbal” (letter-based) *N*-back working memory task for (left) a non-collision-sport control population, and (right) two high school athletes (P213 and P121) before the season (middle column) and subsequent to exposure to repeated head acceleration events, though only one athlete (P213) was diagnosed with a concussion at the time of the second imaging session (rightmost column)**. Activation is depicted for a 2-back vs. 1-back working memory contrast, with preferential activation for the 2-back task indicated by orange–red coloration, and for the 1-back task by blue-cyan coloration.

The PNG study incorporates a fMRI assessment of activation associated with a working memory task (30) as a means to document alterations in the behavior of the brain under a consistent set of demands. Such tasks have proven of marked value in the domain of concussion and other forms of TBI (31–34). To date, the PNG study has focused on use of either singly presented letters (“verbal”) or non-representational line drawings (“visual”) stimuli in a 0-, 1-, and 2-back paradigm (33% target rate). The initial finding reported from the PNG study was a statistically significant relationship in asymptomatic athletes between activation levels associated with differing complexity of working memory tasks and recent mechanical loading, as measured by the number of head acceleration events recorded during the preceding week (35). Subsequent evaluation of the “verbal” task-based fMRI revealed that the extent of the activation alteration increased with exposure, and that the majority of the associated changes are of a “negative” nature – i.e., easier levels of the task are observed to elicit levels of activation more consistent with the difficult levels at Baseline (9, 10). Critically, within the resolution of current functional neuroimaging methodologies, the presentation of neurophysiologic changes in asymptomatic athletes (i.e., those not diagnosed with a concussion) is equivalent to, or possibly even more severe than, that evidenced by athletes who have been diagnosed with a concussion (see Figure 5). Overall, these findings strongly suggest that exposure to subconcussive blows necessitates adaptation by the brain to achieve normal outputs – i.e., brain injury has occurred, which is not necessarily sufficient to preclude successful delivery of information, but does reduce the efficiency of the processing.

Reductions in the efficiency of brain processing are well assessed using various fMRI measures of resting-state connectivity (36–38). SHM-based evaluation using rs-fMRI of athletes reveals that even at the Baseline measurement collision-sport athletes exhibit greater levels of connectivity than their non-collision-sport peers (12), and that this gap decreases (i.e., the Baseline hyper-connectivity decreases) both during periods of relatively high mechanical loading and after extended periods of rest (13). Observation of abnormal connectivity at Baseline suggests that even modest levels of collision-based activity (e.g., summer camps, participation in no-tackling 7-on-7 competitions) may be altering the resting-state behavior of the brain, with collateral pathways assuming a more dominant role at early stages of brain injury. Observed reductions in levels of connectivity during periods of heavy mechanical loading are consistent with other observations of brain injury (11, 39–43). The finding that the connectivity in collision-sport athletes drops down to, but not below, the connectivity levels observed in non-collision-sport controls may provide insight as to why these exposed athletes do not exhibit symptoms – they yet have sufficient connectivity to perform tasks, albeit with greatly reduced efficiency, consistent with the findings from working memory fMRI, above. Of great concern is whether the apparent “recovery” to normal connectivity after an extended period of rest is fully achieved, or if there may be chronic alterations in brain networks due not only a history of concussion (11), but simply due to a history of exposure to repeated head acceleration events.

In recent seasons, the PNG study has incorporated a breath-hold challenge during fMRI (44–46) to induce hypercapnia to evaluate CVR. Research has shown CVR to be an important marker of brain injury severity (47–49), and studies have also shown CVR to be impaired following mTBI (concussion) in younger populations (50, 51) as well as in animal models of mTBI (47, 52). Application of this measurement in the PNG study has demonstrated that the population of collision-sport athletes exhibits at least a transient drop in CVR with the onset of collision activities and requires several months of stable exposure to achieve recovery to Baseline levels (14). Further work will investigate whether this transient population-wide change is representative of all athletes exposed to collisions or if it is primarily driven by those individuals who are exposed to the highest levels (magnitude and number) of collision events.

The various assessments conducted using fMRI combine to suggest that increases in mechanical loading from the current steady-state level – be it rest, or some non-zero level of exposure – produce injury (i.e., altered neurometabolism) that is not immediately reparable by the body. Rather, the brain physiology is altered in a transient manner, followed by a healing response that may or may not produce a return to Baseline levels over a period of several months. Should this hypothesis be correct, it would be wholly consistent with the expectation that an individual who has recently experienced a concussion (i.e., an individual who has accrued enough damage to impair transmission of information to at least one region of the brain) exhibits greater severity of symptoms in response to a second concussion (i.e., further accumulation of injury is likely to result in multiple regions of the brain to which transmission of information is impaired).

### Structural MRI Evaluation

Structural imaging with MRI is used to confirm the structural integrity of, and absence of anomalies in, the various tissues that comprise the brain, specifically the gray matter (neuronal bodies), white matter (axons, glial cells, and associated myelin sheathing), and the vasculature.

T1-weighted morphometric evaluations are a staple of MR studies of neurological development and disorder (53–55) and have revealed changes in the thickness of gray matter regions in athletes who experience repeated head collisions with or without diagnosis of concussions (18, 56). Use of T1w morphometric evaluations within the SHM approach with a growing non-collision-sport athlete control pool will best facilitate detection of structural changes associated with repetitive exposure to subconcussive blows while accounting for the growth of the brain during the ages of the population under study – a key control element that might otherwise confound such analyses.

**Figure 6:**
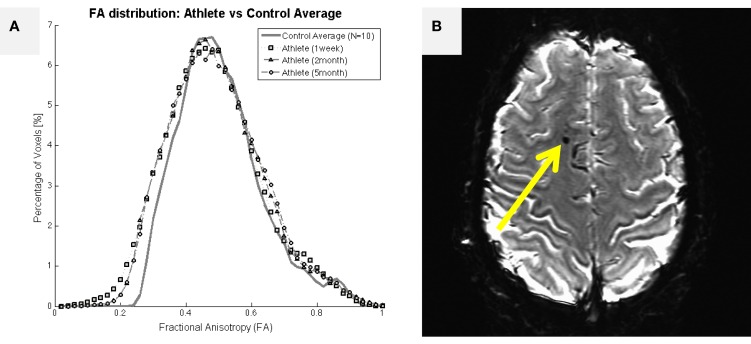
**Structural imaging outcomes for a 19-year-old female athlete who experienced a single traumatic blow to the head, resulting in significant symptoms that have persisted for 2 years, as of this writing**. **(A)** Assessed 1 week after the injury, the female athlete exhibited an appreciable leftward shift in fractional anisotropy distribution relative to a female non-collision-sport athlete population (*N* = 10; solid black line with circles). The deviant distribution remained left-shifted at 2- and 5-months after the injury, failing to return to the normal range. **(B)** Initial T2*-weighted imaging exhibited a marked susceptibility lesion in the frontal lobe (arrow) for this athlete. Subsequent scans reveal a slightly smaller lesion at this same location, possibly indicating a cause for her long-term symptoms.

A more extensive literature exists within the MRI domain on the evaluation of white matter health with DWI in collision-sport athletes (15, 16). In particular, Chun et al. (16) reported that significant fractional anisotropy changes were found in asymptomatic individuals (i.e., those who presumably have not experienced extensive demyelination or damage to glial cells) as late as 3–5 months after the end of their competition season. Individuals who have been diagnosed with symptomatic TBI are well-documented to exhibit large changes in DWI measures, including fractional anisotropy (57–60). Figure 6A presents brain-wide distributions of fractional anisotropy to compare a healthy control population (high school female non-collision-sport athletes) with a 19-year-old female athlete who experienced a significant sport-related concussion, resulting in symptoms that have persisted for 2 years, as of this writing. In this athlete, an appreciable deviation is visible (lower left of plot) 1 week after her injury, with appreciable recovery observable by 2 months post-injury. However, no appreciable shift back toward the healthy population has been observed subsequent to this point in time (e.g., the 5-month post-injury data in Figure 6A are almost perfectly aligned with the 2-month post-injury data). In general, measures such as the distribution of fractional anisotropy tend not to recover to the levels exhibited by the population who has not experienced a meaningful TBI (61, 62). Ultimately, these observations indicate that changes in both concussive and subconcussive injuries are more likely to represent chronic white matter damage, either directly to the glial cells or through demyelination of axons, both of which would be expected to increase the “leakiness” of axons, and reduce fractional anisotropy.

Susceptibility-weighted imaging is the most generally applied methodology to evaluate the presence of damage to the remaining tissue type in the brain, the vasculature. SWI is particularly sensitive to tissue components that have different susceptibility from surrounding structures, with a particular emphasis placed on detection of extravascular deoxygenated blood, largely proposed to be the result of microhemorrhages (63). The PNG study has incorporated a T2*w approximation of SWI. While this approximation provides a reduced sensitivity (and, therefore, a reduced risk of false positives), it reliably detects damage in the brains of those individuals who exhibit persistent symptoms, even after multiple years of recovery time (cf., Figures 1D and 6B). In contrast, four seasons of usage in the PNG study have yet to reveal remarkable findings in the collision-sport athlete population, regardless of the presence or absence of symptoms. However, it must be acknowledged that all of these athletes are, at Baseline, symptom free. While the most sensitive applications of SWI have not yet been incorporated in the PNG study, these findings are suggestive that microhemorrhages may not occur in the absence of particularly severe mechanical stress, and thus may primarily be associated with significant and life-affecting symptoms.

### Magnetic Resonance Spectroscopy Evaluation

Proton magnetic resonance spectroscopy (^1^H MRS) allows evaluation of brain biochemistry, particularly permitting calculation of relative ratios of the concentrations of various metabolites or, with appropriate calculations, the absolute concentrations of metabolites. The measured metabolites are also likely to be of appreciable value in identifying which, if any, of the cell populations have experienced actual mechanical damage – NAA and the combination of the neurotransmitters Glx are likely to directly reflect neuronal health and function (64, 65) whereas the osmotic regulator Ins may best reflect changes in the metabolism of glial populations (66). The PNG study obtains spectra from two regions (8 mL single voxels) in the brain at each assessment: primary motor cortex (M1), due to previous documentation of significant chronic alterations in chemical composition subsequent to concussion (67, 68), and DLPFC, given both its well-documented role in working memory (69) and the initial observations of correlations between changes in working memory fMRI activity levels and exposure to head acceleration events (35). While the general lack of knowledge about the normal ranges for the obtained metabolite concentrations and any variation with growth may reduce the current clinical relevance of these measurements, they are likely to be among the first indicators of altered neurophysiology (i.e., injury). Previous results from the PNG study have revealed substantial variation in these measures during the course of exposure to head acceleration events (19, 20), with some evidence of a lag in recovery to Baseline levels that may persist from one season into the next.

### Discussion of PNG Study Findings

Critically – and consistent with the underlying justification for using SHM as the model for the study – data collected in the PNG study demonstrate that collision-sport athletes do, in fact, accrue injury over the course of the season. Table 1 illustrates how, when using the population mean and 95% confidence interval derived from a corpus of 31 (16 males, 15 females) non-collision-sport high school athlete controls, high school collision-sport athletes participating in the sixth year of the PNG study (2014–2015) exhibit increasing rates of deviant (i.e., outside the control 95% confidence interval) neurological assessment measures as the season progresses from Baseline through the first half (*InSeason1*) to the second half (*InSeason2*), with recovery close to Baseline within 2 months of the end of the competition season (*PostSeason1*) and finally a recovery close to that of the control population finally observed 4–5 months after the end of the competition season and associated accrual of blows (*PostSeason2*).

**Table 1:** **Percentage of neurophysiologic assessment measures in high school athletes falling outside the 95% confidence interval as defined from baseline measurement of a population of 31 non-collision-sport high school athletes (16 males/15 females)**.

Percentage of flagged measurements (%)	Controls	Collision-sport athletes				
	Follow-up	Baseline	InSeason1	InSeason2	PostSeason1	PostSeason2
0	7	0	0	1	1	3
6	10	1	1	0	0	9
13	5	4	3	4	6	8
19	4	8	11	6	8	9
25	0	13	9	11	6	5
31	1	10	7	9	12	1
38	2	3	3	8	4	1
44	0	2	2	3	0	2
50	0	2	2	0	3	1
56	0	0	0	1	1	0

*Underlined values represent the median level of flagging observed for the given population at the given assessment. Data are from the 2014 to 2015 season of the Purdue Neurotrauma Group study. Note that all collision-sport athlete sessions except *PostSeason2* exhibit distributions of flagging rates that are statistically significantly different (*p* < 0.0012; Kolmogorov–Smirnov two-sample test) from that of the *controls**.

The data in Table 1 also raise concerns about the increasingly year-round nature of many youth athletic activities. Even at the time traditionally assumed to represent a “healthy” measurement – immediately prior to the beginning of practice activities – the athletes are, in fact, altered relative to their non-collision-sport peers (*p* < 1.2 × 10^−6^; Kolmogorov–Smirnov two-sample test of *Baseline* vs. *Controls*). This alteration is likely a result of summer practices, summer camps, and participation in summer competitions (e.g., 7-on-7 football tournaments or travel soccer teams). As such, it appears that the athletes are truly closest to being “healthy” (here taken as being most neurophysiologically like their non-collision-sport peers) in the late Spring (February–April), a time at which most Spring practices or seasons now commence for traditional Fall sports (e.g., football, soccer). Even if athletes at the high school level are ultimately returning to a nearly “healthy” level 4–5 months after their competitive season ends (*p* = 0.476; Kolmogorov–Smirnov two-sample test of *PostSeason2* vs. *Controls*), they clearly provide evidence of injury just 2–3 months later, at the beginning of the subsequent season. Ultimately, it is worth asking the question as to whether it is good and healthy to spend 9–10 months per year with abnormal neurophysiology. This question is especially important if one is planning to participate in youth contact sports year after year, often for 10 or more years of prominent development of both body and brain.

A potentially important consideration in addressing the overall duration of time spent by collision-sport athletes with abnormal neurophysiologic behavior is that many of the measures collected (e.g., task-based fMRI, DWI fractional anisotropy, MRS concentrations) have exhibited *team-dependent* variations. These variations are best observed on a population basis, within a given interscholastic team, and are remarkably consistent from one year of study to the next, independent of the number of subjects who participate across those multiple years. These team-dependent trends suggest that it is not simply the number or magnitude of head acceleration events that are experienced that matter, but also the nature and regularity of these events. Teams that experience fewer events or who have more days off per week from collision-based activities also tend to exhibit lower rates of aberrant measures in neuroimaging when compared to their own Baseline measurements (Table 2). Therefore, it is likely relevant in the future to evaluate how coaching style, style-of-play, league and division (e.g., size of schools against whom they compete), and the local population composition are likely to contribute to how readily athletes experience repeated head acceleration events and thereby accrue injury.

**Table 2:** **Comparison of number of full contact activities (e.g., tackling or heading) with the percentage of neurophysiologic measures at each assessment session falling outside the 95% confidence interval defined from baseline measurement within the group of collision-sport high school athletes (*N* = 92; 59 males/33 females)**.

	Contact days/week	InSeason1 (%)	InSeason2 (%)	PostSeason1 (%)	PostSeason2 (%)
Team A (boys’ football)	3	11.0	9.6	5.6	25.2
Team B (boys’ football)	1	8.6	8.8	8.1	26.9
Team C (girls’ soccer)	6	17.5	11.7	11.2	19.0
Team D (girls’ soccer)	5	12.5	13.4	7.3	12.7

*Data are from the 2014 to 2015 season of the Purdue Neurotrauma Group study*.

Therefore, critical questions yet to be asked are when, and how severely are these youth athletes being injured due to their participation in collision-based sports? The answers to these two questions must direct future approaches to prevention and intervention, indicating whether technological modifications/improvements and/or training/educational efforts will suffice to protect the majority athletes, or if more profound changes are necessary to the games and/or culture surrounding them.

Given the observations from Table 1, it appears that by approximately 4 months (but distinctly *not* at 1 month) after the cessation of activities associated with collision-based sports, youth athletes are largely “normal” in measures of neurological behavior and biochemistry. Such observations – apparent in the PNG data across multiple seasons – strongly argue that the process of neurological repair requires anywhere from 5 to 12 weeks of “rest” to overcome the damage brought about by a season’s worth of participation.

Of particular interest, it would appear that the PNG study has documented that athletes are *not* at full health at the end of the summer – immediately prior to the beginning of “full” contact practices – as is traditionally assumed in prospective studies (70). Rather, the “limited” contact practices that tend to take place in the summer period (between academic years) likely involve sufficient head acceleration activity that some brain changes have already been set in motion. This interpretation is consistent with the marked increase in *PostSeason2* flagging rates in Table 2, wherein the collision-sport athletes are compared to themselves at the Baseline measurement, coupled with the *decrease* in flagging rates at *PostSeason2* in Table 1, wherein the collision-sport athletes are compared to the non-collision-sport controls. The deviations associated with the Baseline measurement seem to be most apparent for the biochemistry and brain network measures obtained by the PNG – cf., Poole et al. (19, 20) and Abbas et al. (12, 13). Additionally, it appears that even *within* the duration of participation, some level of repair is ongoing as athletes tend to stabilize in many measures within the season, only to exhibit a further drop when the level of exposure/participation undergoes a relatively abrupt increase (e.g., at the beginning of “full” contact practice; as teams prepare for post-season play). See, e.g., Svaldi et al. (14); Abbas et al. (12, 13).

Regarding severity, we hypothesize that the injuries accrued in these asymptomatic athletes due to “subconcussive” events are not appreciably different from those experienced by “concussed” athletes. Over the first 6 years of the PNG study, the 12 symptomatic athletes were (on average) flagged to a greater degree across the ensemble of measures, but this difference has not been found to be statistically significant. Given that athletes diagnosed with a concussion are subsequently held out of participation and, therefore, experienced a period of markedly reduced exposure to acceleration events and during which healing may occur, it should perhaps not be surprising that the majority of acquired measures have not indicated a statistical difference between athletes with or without a history of concussion, be it recent or long-term. Ultimately, there are likely to be consequences for athletes who are “Altered,” albeit not identified as “Injured,” but these are most likely to occur under physical duress or as limitations in the ability to concentrate or focus for extended periods of time. While, theoretically, coursework would be expected to suffer, the majority of athletes receive significant support during their competition seasons, potentially obscuring any transient deficits that may exist.

It is important here to note that the PNG study has found evidence that refutes the argument that within-season changes in fMRI measures are the consequence of substance use by the athletes, whether before, during or after the season. For one season (2013–2014), the PNG study incorporated a urine dip card drug test in 124 neurological assessments, conducted on 32 high school collision-sport athletes (16 boys’ football and 16 girls’ soccer). Using a dip card drug test (uVera Diagnostics) for 12 drugs – amphetamine, barbiturates, benzodiazepines, cocaine, Ecstasy (MDMA), marijuana (THC), methadone, methamphetamine, opiates, oxycodone, phencyclidine, and propoxyphene – a total of six tests (~4.8%) were found to exhibit a positive result: three for marijuana (two football athletes), two for Ecstasy (two football athletes), and one for amphetamine (one soccer athlete). This level of substance usage is consistent with the 2013 National Survey on Drug Use and Health which reported illicit drug use by 8.8% of youths ages 12–17, which also found marijuana use to be most common (71).

## Prospective Detection and Prevention of Concussion

### Validation of Protective Equipment

One of the greatest benefits of SHM using imaging will be the ability to quantitatively validate novel procedures or equipment that are intended to produce greater brain health. At present, a large number of products are currently being marketed with claims of enhancing safety, but for which evaluations are predicated on outdated or inappropriate testing procedures. The prospective feedback associated with the SHM process, particularly when combined with neurophysiologic assessments, can permit the claims from these and future advances – be they equipment, training techniques, or therapeutic agents – to be verified or refuted in the target subject populations under meaningful field conditions.

The deleterious effects of head impacts in sports were noticed over 100 years ago, and there have been methodical advances in safety since that time, though notably without the inclusion of an SHM approach. In 1906, the National Collegiate Athletic Association (NCAA) was formed after a series of conferences held at the behest of President Theodore Roosevelt in order to effect changes in college football. The original goal of the organization was to eliminate the massive head traumas that had caused 18 deaths and many more catastrophic injuries during competitions in 1905 (72). A series of rule changes and, in 1939, the requirement that players wear helmets, dramatically decreased the occurrence of these injuries. Initially those helmets were made of leather, lacking facemasks, but evolved over time. Plastic helmets with chin straps were constructed in 1940, and the facemask was introduced in 1954. Cushioning systems evolved from suspension assemblies to polyurethane foam padding and air bladders but improvements in design were guided only by limited biomechanical testing (73–75).

The National Operating Committee on Standards for Athletic Equipment (NOCSAE) was established in 1969 and introduced a drop test-based criterion for certifying football helmets in 1973. At the time, it was based on the best science available, most of which was gathered at Wayne State University, and it reduced the number of skull fractures dramatically. Unfortunately, the standard still fails to address the mitigation of TBI, and it is clear that the current helmets are not providing sufficient protection for the current style-of-play [e.g., Ref. (13, 19, 35, 76)].

With the valuable insights that the SHM model has provided to our current understanding of TBI, the effect of repetitive head impacts, and the recovery process, it is fruitful to “close the loop” and utilize SHM to reduce the burden of head injuries across all the at-risk populations, starting with contact sports. Utilizing MRI, fMRI, and MRS as our gold standards of neurophysiological health, it is now possible to quantitatively evaluate the effects of many different interventions and develop new standards that take advantage of modern medical imaging methods. Longitudinal studies such as those described herein provide the context for determining whether a particular athlete’s brain has statistically deviated from the pre-season Baseline. Any intervention that significantly decreases the number of athletes whose neurophysiology is altered could therefore be considered for future development.

There are many possible interventions. Reducing or eliminating hitting in practices could potentially decrease the number of head impacts by more than 50% for most high school and collegiate athletes [e.g., Ref. (77)]. Similarly, tracking the number of head impacts using telemetry-based sensor systems may make it possible to set limits on cumulative exposure that integrates the number and magnitude of the hits. Setting a limit on the number of acceleration events or their cumulative magnitude and then repeating the PNG study would make it possible to determine whether that level was sufficient to provide better protection to the athletes. Perhaps more importantly, there is a fundamental need for better protective equipment including helmets and their continued development would fit well with an MRI-based SHM approach that also incorporates the current NOCSAE standard. Introduction of improved technologies can also be evaluated using MR-based imaging. Interestingly, SHM provides the means to make the helmets themselves “smart” in the sense that they could potentially be self-evaluating, alerting the appropriate officials when the helmet is no longer serviceable.

### Validation of Monitoring Equipment

Early work by Pellman et al. (78) suggested that concussions were a result of head accelerations averaging approximately 98 g, but there was a flaw with their study that has caused a fundamental misconception about head injuries to persist to the present day. Beginning with the assumption that concussions are only caused by a single blow, they obtained video of head impacts that immediately preceded concussions during game situations and attempted to recreate those collisions using Hybrid III test dummies. While they examined the concussed and non-concussed players involved in the collision, they did not recreate a sufficient cross section of hits that occurred without causing concussions. If they had, they would have realized quickly that blows in the range of 60–120 g are quite common (79) and that the frequency at which they occur virtually requires that neural damage accumulates with each such impact, not only those that were directly associated with diagnosed injuries.

Since then, various studies have examined head impacts in both high school and collegiate football (35, 79–85). The average values tend to vary between 20 and 35 g depending on the sensor system used and the setting for the minimum hit threshold. For instance, the HITS consists of six uniaxial accelerometers distributed around the crown of the head and records an impact any time the resultant linear acceleration at the center of mass exceeds approximately 10 g. In contrast, the xPatch system, consisting of a triaxial accelerometer and triaxial gyroscope, can record impacts using a range of linear acceleration thresholds, commonly chosen to be a value between 10 and 20 g.

Perhaps more importantly, a typical distribution of hits decays monotonically from 20 to 120 g, and those distributions are virtually identical whether or not the athlete was diagnosed with a concussion. It should also be noted that hits above 200 g have been observed with the highest PNG study recorded acceleration event to-date being 289 g (79). Under the Pellman model, a hit over 185 g would cause a concussion 99.9% of the time, but between 2009 and 2013 PNG recorded 27 such blows (out of 80,000 total head impacts), with none of these events resulting in a diagnosed concussion. Conversely, no blows exceeded 120 g (peak = 116.5 g) among the 10 blows immediately preceding each of the eight concussions observed in the PNG study to-date in athletes for whom preceding HITS telemetry data exist. In fact, only 3 of these 80 blows exceeded 80 g (average = 31.9 g). Taken together, these data indicate that the past tendency to observe larger blows as being the “cause” of concussions [e.g., Ref. (80, 81)] is not, in and of itself, sufficient to rule out the potential risks associated with repeated subconcussive blows to the head. Rather, appreciable variability of the outcome of large blows to the head – i.e., that no clear relation has been observed between the magnitude of the “causative” blow and the subsequent clinical severity – is instead suggestive that sub-concussive blows do play a meaningful role in the accumulation of damage.

Telemetry-based studies have also shown that the number and magnitude of head impacts vary significantly with position (82, 86, 87), particularly of interest given that PNG has observed that neurophysiological changes also exhibit a positional dependence – i.e., primarily offensive/defensive linemen exhibiting neurocognitive changes in the absence of symptoms (35). These results strongly suggest that monitoring and limiting the number of head impacts have the potential to decrease the overall burden of neurotrauma in sports, but it should be noted that the current systems have enough measurement error, and there is at least as much uncertainty in the damage thresholds for each individual, that monitoring head impacts alone cannot provide sufficient safeguards.

### Application to Predictive Modeling for Prevention of Injury

Accepting the engineering model that the presentation of symptoms (i.e., diagnosis of a concussion) is the consequence of accumulated injury debunks the myth that there need necessarily be a particular “threshold” beyond which a singular acceleration event to the head (whether due to a blow or whiplash) will automatically produce a concussion. Rather, the inability of researchers to find such a threshold actually argues strongly in favor of the accumulation of injury as the cause of the vast majority of diagnosed concussions in athletes.

**Figure 7:**
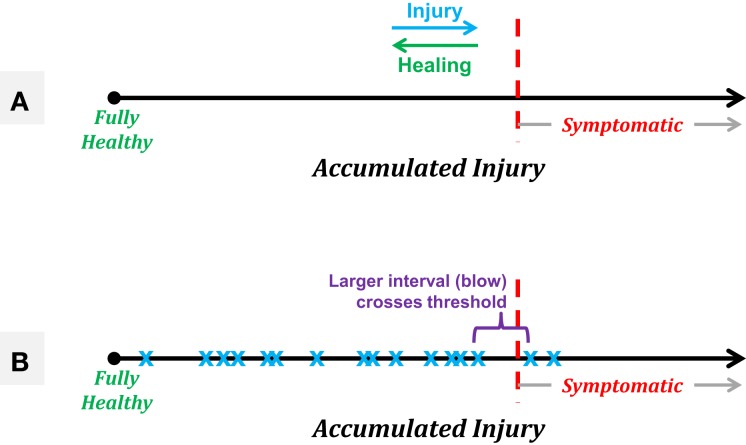
**Schematics of a linear model of accumulated injury based on the concept that it is not necessarily a single blow that induces symptomatic injury, but rather the summation of a series of smaller injuries that, in and of themselves, would not be expected to produce symptomatic outcomes**. **(A)** Treating accumulated injury as the *x*-axis, an athlete starts “fully healthy” at the leftmost point, with an unknown threshold of injury along the axis, beyond which the athlete will exhibit symptoms associated with a diagnosed concussion. Healing processes may take place between sequences of blows, resulting in gradual recovery to the left, while each experienced acceleration event will push the athlete to the right. **(B)** A simple example of the random incidence paradox in which a series of subconcussive events (blue “x” markers) eventually accumulate such that the athlete moves into the symptomatic region. The “causative” event is expected to be of a magnitude equal to twice the average experienced event, and would not be expected to produce symptoms on its own.

A model of injury development can help clarify this potentially controversial statement. Consider the accumulation of injury as a marker that moves along a single linear axis, running left to right with “fully healthy” representing the leftmost end of the axis (see Figure 7A). Each experienced blow will move the athlete slightly further to the right, with the distance traversed being a function of the severity of the blow (a function dependent in part on the linear and rotational acceleration and likely the location, as well). Assuming the existence of some “threshold” along the axis beyond which the athlete will exhibit symptoms, and continued accumulation of events in the absence of rest or repair (see below), the athlete will eventually exceed this threshold and exhibit symptoms. This linear model illustrates why efforts to characterize the event “causing” a concussion tend to observe highly variable incident forces across athletes, yet also to observe ensemble linear acceleration averages (80–90 g) that are highly consistent across evaluations [e.g., Ref. (78)]. Specifically, this model can be used to illustrate the *random incidence paradox*, drawn from probability and operations research (88), which is encountered frequently in everyday life, particularly as one waits for (as an example) a bus. Specifically, this paradox states that the time interval between the arrival of the preceding bus (i.e., that which left before the observer arrives at the stop) and the arrival of the next bus will be *twice* the average (expected) interval between bus arrivals. As applied here to the concept of injury, the random incidence paradox informs us that we are more likely to cross an underlying threshold (e.g., of injury) with a large event than with a small event (see Figure 7B).

Note that accepting this model and hypothesis does not equate to stating that any individual can be exposed to any level of acceleration without necessarily being injured – in fact, it is quite the opposite. This model assumes that each and every acceleration event experienced produces some level of neurophysiologic injury, but that the breakdown of the transmission of information within the brain does not occur until some threshold has been crossed. Critically, this threshold is expected to vary across individuals, and likely be different for various regions in the brain – e.g., the limbic cortex, residing just above the rough lower surface of the brain cavity, is likely to have a markedly different tolerance for acceleration events than would the parietal lobe. Therefore, using fixed thresholds of event acceleration or even fixed thresholds of accumulated acceleration are likely to produce large numbers of athletes who are pulled out of participation for examination while yet asymptomatic, and lead to a false sense of security that athletes who have not been exposed to those specific levels are “healthy.”

The accumulation model of injury may also be extended to account for repair processes. Evidence from the PNG study suggests that athletes who are allowed more days off between activities involving repeated acceleration events (i.e., practices and games) generally exhibit fewer deviant measurements in neurological assessments than those athletes who participate in such activities on an almost daily basis (cf., Table 2). In the context of the linear model introduced above, this simply means that after each activity (and possibly after each single event), normal physiologic healing processes move the athlete back to the left (i.e., toward “fully healthy”), making it less likely that the next blow will prompt a failure in the network. These healing processes allow for a symptomatic athlete to return to an asymptomatic state or for an athlete who evidences alteration to be less likely to experience a failure in brain networking. Further longitudinal studies using SHM must be conducted, however, to evaluate under which conditions an athlete can truly return to the origin of this line. Our current work suggests that there exist conditions under which a return to full health cannot be achieved. Interestingly, the guidelines for use of ImPACT™ (used by the NFL, most colleges, and most high school athletic associations) support this suggestion. The battery is conducted during pre-season activities to obtain a baseline value, and is then used to re-evaluate athletes subsequent to a diagnosed concussion, specifically to determine if they have reached a state statistically similar to their baseline. However, once the athlete’s performance has returned to be within the “reliable change index” of the baseline measurements, this new value is to be treated as the baseline for subsequent evaluations. In the absence of marked learning of the tasks, this approach will be biased toward the new baseline being below the original – implicitly assuming that athletes will not return to their original cognitive norms.

Using SHM in conjunction with the accumulation model of injury, it is feasible to *increase* the ability of athletes to participate in collision-based sports of their choosing while *decreasing* the short-term risk of injury – i.e., *more* athletes could play *more* sports, *more* safely. Long-term application of an SHM approach should make it feasible to construct a predictive model that can assess the risk of an athlete for the exhibition of either deviant measures in a neurological assessment (e.g., neuroimaging, neurocognitive testing) or symptoms subsequent to a next acceleration event or predicted series of next acceleration events. This predictive model would presumably incorporate measures of pre-season Baseline neurophysiologic behavior with an actively monitored history of exposure to acceleration events to create an athlete-specific prognosis at any given point in time. Such a model could be useful in identifying athletes for whom it would be advisable to skip a practice in order to exhibit a (substantially) reduced risk of either neurophysiologic alteration or injury after the following game. While a model for youth athletes is presently only speculative, models of a similar nature are currently used in a number of engineering applications and provide a framework for future efforts to encourage greater engagement in youth sport.

## Evaluation of Current Concussion Research

Even when a concussion has been identified, meaningful assessment of the prognosis remains elusive. Current studies such as the $35 million NCAA–DOD CARE Consortium and the $60 million GE–NFL Head Health Initiative are focused on assessing the outcomes (the so-called “natural history”) of concussion based on biomarkers, in large part to be obtained from MRI. Improved prognostic post-injury assessment with MRI will, in fact, offer a substantial benefit over the current standard-of-care following a concussion, namely the use of x-ray or CT. While these traditional tools are generally effective for detecting skull fracture or subdural hematoma, other forms of damage such as small aneurysms or “leaky” vessels are unlikely to be observed and may lead to catastrophic outcomes similar to or worse than those experienced by Cody Lehe (see above). The lack of pre-season Baseline assessments in these studies will, unfortunately, significantly limit advances in our understanding of the etiology of concussion. Moreover, any benefits obtained from these MRI-based studies will only apply to those athletes who already exhibit symptoms, and are thus likely to be examined for injury.

The presence of comparable neurophysiologic changes between symptomatic and asymptomatic athletes highlights the fundamental weaknesses of the ongoing multi-center studies. While these studies will develop the natural history of (here referring to the recovery from) concussion by imaging multiple groups – e.g., concussed individuals, including collision-sport athletes; collision-sport athletes matched with concussed individuals in a variety of categories, but who have not been diagnosed with a concussion; and non-athlete controls matched to concussed individuals in gender and academic factors – in a longitudinal manner, there will not be a systematic assessment of the athletes pre-injury. The difficulty in identifying injury only on the basis of symptoms was starkly revealed in Talavage et al. (35) with the observation of appreciable neurophysiological changes in youth (high school) collision-sport athletes who were asymptomatic and, therefore, never examined or diagnosed as being “concussed” by their team’s health-care professionals. Data from the PNG study and elsewhere [e.g., Ref. (11, 15)] demonstrate that simply because an athlete has not been diagnosed with a concussion does not imply that said athlete is “healthy,” nor that that athlete’s brain will appear comparable to the brain of a non-collision-sport athlete (cf., Table 1), let alone to the brain of a non-athlete. Therefore, given that the intended collision-sport control group will have experienced a comparable level of exposure as the concussed collision-sport group, it is highly probable that only subtle differences will be observed, and these may be more likely to represent a distinction between significant, lasting injury (e.g., see Figures 1D and 6A) and transient, recoverable injury – i.e., they will (as intended) have prognostic value, but be of extremely limited benefit with regard to treatment.

That these significantly funded multi-center research efforts focus solely on the *outcomes* of a disease/disorder, and are not investigating its etiology or course of development, should be of great concern to the medical community. Entire fields of clinical medicine rely on early detection of biophysical changes that are not, in and of themselves, dangerous, but do serve as indicators of increased risk for development of long-term disorders profoundly affecting patient quality of life – fundamental examples of SHM approaches to medicine. Simple examples include chronically elevated blood sugar levels, now interpreted as a “pre-diabetic” condition; and elevated blood pressure and cholesterol levels, indicating increased risk of cardiovascular disease. While it is not uncommon for segments of the medical community to resist recognition of abnormal physiologic behavior as a “disorder” or “disease” if no treatment exists – e.g., celiac disease (89) and the linkage of *H. pylori* to ulcers (90) – the lack of such recognition does not reduce the associated suffering. Just as thousands (if not millions) ultimately suffered more significantly from such unrecognized disorders prior to medical recognition, we must now ask how many thousands (if not millions) of youth must suffer from neurological damage in the short- and possibly long-term simply because our collective definition of “concussion” is incomplete, and only captures those symptoms associated with near-complete failure of neural circuits to communicate information among themselves.

The lack of a Baseline (pre-exposure) measurement in the prominent multi-center studies will further be exacerbated by an appreciable lack of knowledge regarding the history of exposure to head acceleration events for the subjects being assessed. Among the general populace (e.g., the anticipated patient populations in the GE–NFL study), it is extremely rare for documentation to exist of the nature, direction and associated force of a blow that prompts care for TBI. And while it was the original intention of the NCAA–DOD CARE Consortium to exploit telemetry to monitor blows to the head experienced by the athletes, the initial season of study will not use any telemetry system for non-football athletes, and football athletes will be monitored using HITS – a system documented to have sufficient limitations regarding accuracy (91) that the PNG study generally restricts usage of these data to counts of relevant events in coarse groupings (e.g., “above 60 g”).

The lack of accurate acceleration event information will be most unfortunate, as there is a gradual growth in the acceptance that, somehow, exposure to head acceleration events or collisions may decrease the resistance to injury from a subsequent blow. Ultimately, this change in apparent injury threshold is informative that either the biochemistry or the cellular integrity of the neural system is being altered by these preceding events. And while some alterations in biochemistry truly can be temporary in the associated impairment (e.g., temporary threshold shifts after exposure to sustained periods of loud noises), many of these debilitating changes will become permanent if the offending environment is not corrected or avoided (see, e.g., limits on occupational noise exposure). There does exist a class of biochemical changes that alter “threshold” functionality in an arguably positive or protective manner, highlighted by how damage to cellular structure serves as the basis for redevelopment/reformation of certain tissues, such as bone and muscle. It should be noted, however, that the repair mechanisms in the brain differ considerably from the musculoskeletal system. Bone fractures go through a process of callus formation, mineralization, and bone adaptation, typically resulting in a stronger structure. In contrast, glial scars in the central nervous system act to prevent further damage, but ultimately prevent axons from reforming connections across the scar.

Extending such large-scale research efforts through the integration of baselined MRI-based imaging assessments will not only provide greater sensitivity and specificity for diagnosis but also enhance our ability to prevent such injuries through improved equipment, training, and monitoring (92). Given the weaknesses outlined above, it should be a legitimate concern among researchers in this field that the large scale and funding levels of the greatly overlapping NCAA- and NFL-supported studies have conveyed a sense to both the general public and federal and state granting agencies that the problem of concussion in sports (including youth sports) is well-addressed. However, while substantial funding does now exist for the study of how athletes recover from the diagnosis of concussion, there is (by comparison) extremely little money being directed at understanding how the associated symptoms arise.

Therefore, it is critical that the scientific and medical community shift their focus and direct funding into the characterization of pre-symptomatic injury, ideally using an SHM framework, as this is the best and fastest pathway by which truly *preventative* measures may be developed and properly evaluated. Moreover, it is the most efficient method for developing and testing potential *treatments*. Both can be accomplished by proposing modifications to equipment, training or interventions, then implementing these modifications and ultimately documenting that the *pre-symptomatic* changes are correspondingly mitigated, thus reducing the overall burden of neurotrauma.

## Conflict of Interest Statement

The authors declare that the research was conducted in the absence of any commercial or financial relationships that could be construed as a potential conflict of interest.
